# Scarce Resource Allocation for Critically ill Patients During the COVID-19 Pandemic: A Public Health Emergency in São Paulo Brazil

**DOI:** 10.6061/clinics/2021/e2191

**Published:** 2021-01-11

**Authors:** Chin An Lin, Juliana Bertoldi Franco, Sabrina Correa da Costa Ribeiro, Luciana Dadalto, Leila Suemi Harima Letaif

**Affiliations:** I Medicina Internal Geral. Comite de Bioetica, Hospital das Clinicas (HCFMUSP), Faculdade de Medicina, Universidade de Sao Paulo, Sao Paulo, SP, BR; II Departamento de Odontologia, Hospital Auxiliar de Suzano. Comite de Bioetica, Hospital das Clinicas (HCFMUSP), Faculdade de Medicina, Universidade de Sao Paulo, Sao Paulo, SP, BR; III Emergencias Clinicas, Hospital das Clinicas (HCFMUSP), Faculdade de Medicina, Universidade de Sao Paulo, Sao Paulo, SP, BR; IV Centro Universitario Newton Paiva, Belo Horizonte, MG, BR; V Instituto Central, Hospital das Clinicas (HCFMUSP), Faculdade de Medicina, Universidade de Sao Paulo, Sao Paulo, SP, BR

As granted by the Federal Constitution, in article 196, Sistema Único de Saúde-SUS (Brazilian Unified National Health System) is an in-depth public health system with universal coverage in Brazil. The Constitution declares health as a fundamental right of all citizens and every person who is inside Brazilian national borders, and that it is an obligation of the State to ensure that everyone has access to the public health system (1).

Normally, public resources struggle to meet the health needs of all individuals, since these demands are huge and public resources and facilities are not up to par (2,3). The results are that ill people are under-assisted in primary and secondary health care centers, and tertiary hospitals.

The relationship between the public health demand and the public health resources is not balanced. Thus, the Brazilian Public Health System is in an everlasting deficit. There is always a lack of personnel, medical devices, health care services, and so forth (4). There is also an increasing number of lawsuits to force SUS to acquire medications not available in its list of medications approved by Agência Nacional de Vigilância Sanitária (ANVISA) (National Sanitarian Surveillance Agency), which notably is expensive medication (5). Therefore, it is always difficult to apply bioethical principles (beneficence, non maleficence, autonomy and justice) to provide a better distribution of public resources assisting people in need. The fair allocation of resources to assist more people or to assist severely-ill people with expensive medications is a prevailing challenge in the national scenario.

Therefore, in times of great public health emergencies like the COVID-19 pandemic, the fair allocation of resources has become a critical attempt. Is someone more deserving than someone else in terms of ensuring their own survival? Such questions must now be considered as the pandemic continues to affect more people every day and there aren’t enough intensive care beds for every severely ill person who requires it.

In the backdrop of the COVID-19 pandemic, intensive care resources are in high demand but are not always available. In addition to the great demand for intensive care beds, the long occupation of intensive care units by severely-ill patients in intensive care units and specific mechanical ventilation methods, creates further challenges in availing more intensive care beds and the reallocation of used beds to new patients. How does one decide who has more priority to receive intensive care and invasive mechanical ventilation assistance (6)?

This manuscript aims to discuss the important issue of intensive care allocation during the pandemic in the main reference center for COVID-19 in São Paulo (the most affected site), Brazil. We do not focus on the technical question of the criterion per se and whether the criterion worked, but on the discussion of the bioethical implication of adopting the criterion.

Emmanuel et al. (7) foresaw and stated the need to allocate resources rationally, including individual protective gear, ventilators, and hospital beds to face the pandemic. However, he pointed out the need to consider the ethical value of these measures. In many parts, the article illustrated a scene of what would happen with countries facing this public health emergency.

Emmanuel drew attention to the fact that when one tries to ration scarce resources when facing a public health emergency, many bioethical values should be considered, such as the following:

1) Maximizing benefit: saving the most lives and saving most life years: maximize prognosis;
2) Treat people equally: random selection among people with similar prognosis;
3) Promote and reward instrumental value (benefit to others):
a) Retrospectively: prioritize resources to those who have made relevant contributions (give priority to research participants and health care workers when other factors such as maximizing benefits are equal);
b) Prospectively: prioritize resources to those who are likely to make relevant contributions (give priority to health care workers).
4) Give priority to the worst off:
a) Sickest first (used when it aligns with maximizing benefits);
b) Youngest first (used when it aligns with maximizing benefits such as preventing spread of the virus) (7).

Taking into account these bioethical considerations in order to establish an objective parameter to help medical staff make decisions about heading severely ill patients to intensive care services, the emergency service staff decided to search for technical and objective parameters, based on bioethical principles, to screen the patients that are to be directed to the intensive care unit (ICU).

We proposed a criterion for allocating critical patients to the ICU of Hospital das Clínicas, the teaching hospital of the Faculdade de Medicina da Universidade de São Paulo (the University of São Paulo Medical School), which is a tertiary health center with approximately 2400 beds and is considered to be the largest public hospital complex in Latin America.

Before the pandemic reached the state of São Paulo, the hospital administrative council decided to transform - equip the “Instituto Central” (a nurseries and intensive care units institute) and enable its health care team (including physicians, nurses, dentists, physiotherapists, pharmacists, medical residents, and administrative staff) - into a reference institute to care for severe COVID-19 patients referred from the city of São Paulo (with approximately 10 million inhabitants). For this purpose, 500 beds in the nursery and 300 beds in ICU were provided. Furthermore, other specialty wards and beds in intensive care that were not directly involved in confronting the COVID-19 pandemic and were originally located in the “Instituto Central” were shifted to other institutes located in nearby buildings. They continued to take care of no COVID-19 patients, including the emergency room, nursery, and ICU. This operation involved thousands of people and was performed before the pandemic struck. All processes were conducted to ensure that patients with other diseases continued receiving assistance by observing social distancing with COVID-19 patients or suspected COVID-19 patients.

As a tertiary health service, Hospital das Clínicas was the main health center attending to the most serious and complex patients from other health services. As the number of requests for COVID-19 transfers in São Paulo to Hospital das Clínicas grew, an emotional, moral, and bioethical issue was raised. Which patient should be sent to the intensive care bed immediately, and which one should wait? Besides technical issues presented by the unavailability of ICU beds to meet all the demands at the same time, there was also a bioethical concern involved in identifying who would have a greater probability of survival and also the construction of a shared decision with the family or the patient’´s representatives. The shared decision involves informing the patient and his representative of the real probability of survival and providing support to search for and decide upon the best treatment - either through intensive care, with all types of aggressive and invasive treatment (and the consequent physical and psychological suffering it may represent), or by forwarding to nursing or palliative care.

To provide an objective instrument to serve as a parameter, but not as the only tool to help emergency service personnel in making decisions, an emergency service team translated (from English to Portuguese) the University of Pittsburgh Executive Summary Allocation of Scarce Critical Care Resources During a Public Health Emergency (8). This summary contemplates a wide range of clinical parameters of severity and foresees the probability of survival during and after ICU permanence by considering the chronic morbidities that the patients may have. Therefore, the Committee of Bioethics of Hospital das Clínicas of the University of São Paulo (CoBi-HCFMUSP) were asked to consider its bioethical aspects before establishing it as a parameter for decision making in the emergency room. The parameter was based on the Sequential Organ Failure Assessment (SOFA), with scores varying from 1 to 8, plus variables based on previous chronic diseases that could potentially compromise the patient’s survival (Table 1). Patients with a smaller score should be allocated to the ICU to receive mechanical ventilator assistance (9). This would be an exceptional situation, only carried out in the case of an absolute lack of ICU beds. 

This criterion was applied to all patients forwarded to Hospital das Clínicas from primary and secondary care centers around the city of São Paulo before their admission to the hospital.

The main concern of establishing such a parameter is the bioethical conflict (7). Besides the problem of not making the optimum effort to preserve patients’´ lives (beneficence) after scoring their chances of survival and eventually not forwarding them to an ICU bed, there also arise serious legal problems according to Brazilian laws: it is illegal for a health care professional to not deliver proper means to safeguard patients’ lives. Brazilian laws and even the Medical Ethics Code reinforce physicians to do everything to safeguard the patient’´s life, regardless of the patient’´s desire to die with dignity or refusal to continue futile treatment. Recently, Conselho Federal de Medicina (CFM - Federal Council of Medicine) published a resolution on the refusal of treatment (10), where it states that every capable patient (full aged, conscious and not hindered by any clinical conditions of expressing free will), could refuse undergoing any treatment, unless at risk of death. In this case, physicians should deliver any means to preserve the patient’s life, denying his right to refuse any treatment. Thus, deciding not to forward a critically ill patient (even with his consent) to ICU based on a scores scale (even submitting a patient to a very painful and invasive experience with intensive care, with minimal or null chance of surviving) could generate not only bioethical conflict, but also legal issues.

To minimize possible bioethical conflicts and decrease moral and ethical stress in the frontline emergency service team, the Bioethics Committee at the Hospital das Clínicas first considered the adopted objective parameter as necessary in an exceptional period where resources are scarce and the choice of who would benefit from these resources cannot be a random choice and on a first come, first served basis. The chosen parameter was SOFA (to save life) (11) plus assessment of underlying chronic medical conditions that could seriously compromise near-term survival even when the patient survived the acute condition imposed by COVID (to save life years) (12). Although there is no unfailing parameter that is totally reliable, the adoption of such a criterion undoubtedly relieves the emergency service team from moral and ethical stress, since in an unprecedent time like the current pandemic, the main fear is being unable to providing the right resources to the right patient.

Therefore, allocating scarce resources to those who have better conditions to survive is justifiable in a public health emergency. It is clear that everyone has the right to total access to public health resources granted by the Brazilian Constitution (1), and the choice and consequent designation of certain patients to ICU cannot be done authoritatively. The decision should be widely discussed, the family informed and the decision shared after intense negotiation. Moreover, the decision cannot be made irresponsibly, and should be first considered by the staff that oversees the ICU bed requirement. They must inform the patient, or his/her families or representative(s), of the availability of ICU beds before the patients’ arrival. After the patient’´s arrival, along with their clinical evolution and evaluation, families and representatives should be approached again to make a shared and transparent decision. The shared decision is not a warranty of a successful outcome of a treatment, but to ensure the participation of all parties involved in reaching an acceptable decision for each concerned party (13). As a shared decision is based on the respect for the autonomy of each concerned party, it should not be based on the sole authority of any one party; yet, in a practical sense, that is very hard to achieve.

The allocation of resources to serve the most fit to survive is not an acceptable argument to most families, nor us. However, if a patient’s score is worse than the adopted standard, it means, in addition to having less chance of survival (11), that the patient would be subjected to harsh clinical measures in ICU, often in dysthanasic conditions (to die with suffering) or suffering (maleficence) (14). Thus, patients in this condition should be spared and not enrolled in ICU. Obviously, all kinds of care concerning comfort to the patien’s suffering such as analgesia, support for dyspnea, and sedation should be assured. Effective communication with patient’s as well as their families and representatives is essential. We should remember that technical and objective parameters are tools to help make decisions; however, they are only a few among many aspects that should be taken into account. The most important aspect is shared decision making. Only through the consensus of each party can we make better decisions for delivering the right care to patients.

## CONCLUSION

In times of a pandemic, some issues that were not a part of our daily thoughts emerged to demand proper reflection. We are not sure of the outcome of adopting this criterion, but it is thought to be one of the practical tools for the fair allocation of ICU beds.

Certainly, questions like beneficence, non-maleficence, autonomy, and justice, that are considered to be core bioethical principles, are constantly in evidence in everyday health care practice. During, an unprecedented public health emergency like the current pandemic, these principles emerged as acute questions. How to deliver the best care to every patient in need is the main objective, although with limited beds and personnel, this objective is not always possible to reach.

The adoption of an objective criterion was an attempt to meet this goal. As an ongoing practice, in spite of not having data to measure its outcome, it certainly helped the emergency service team by easing the burden of selecting patients based on a criterion. At the same time, this experience promoted a deep reflection on bioethical principles, and hopefully it can help to promote a better view on bioethical principles in the ongoing pandemic as well as in the daily health care practice excluding public health emergency situations.

## AUTHOR CONTRIBUTIONS

Lin CA wrote the manuscript. Franco JB reviewed the manuscript and contributed to improve the manuscript. da Costa Ribeiro SC and Dataldo L proposed the objective criteria on which the manuscript was based. Letaif LSH reviewed the manuscript.

## Figures and Tables

**Table 1:** Strategy adopting several variables to allocate intensive care beds during a public health emergency.

Main Goal	Specification	1	2	3	4
To rescue more patients	Short Term Survival Prognosis (SOFA*)	SOFA<6	SOFA 6-9	SOFA 10-12	SOFA>12
To ensure more time (years) of survival	Long Term Survival Prognosis (Medical evaluation of comorbidity)		Important comorbidities with substantial impact on long term survival		Severe comorbidities, estimated survival less than a year.

SOFA* Sequential Organ Failure Assessment.

Scores varying 1-8. Patients with a smaller score and lower comorbidity should have priority to receive intensive care beds.

**Examples of important comorbidities (significantly compromise a long-term survival)**

• Moderate Alzheimer’s disease or moderate dementia

• Neoplasm with life expectancy <10 years of survival

• Class II heart failure New York Heart Association (NYHA)

• Moderate chronic lung disease

• Terminal renal disease in patients aged <75 years

• Coronary diseases with several arteries compromised

• Hepatic Cirrhosis with episodes of decompensation

**Examples of severe comorbidities (significantly compromise a short-term survival <1 year)**

• Severe Alzheimer’s disease or severe dementia

• Neoplasm in palliative care (including palliative chemotherapy or radiotherapy)

• Class IV Heart Failure New York Heart Association plus fragility

• Chronic Obstructive Pulmonary Disease plus fragility

• Cirrhosis with MELD ≥20, ineligible for hepatic transplantation

• Terminal renal disease in patients >75 years
